# Trimodal Multiplexed Lateral Flow Test Strips Assisted with a Portable Microfluidic Centrifugation Device

**DOI:** 10.1021/acs.analchem.4c02432

**Published:** 2024-09-14

**Authors:** Man-Wen Wang, Zong-Min Chen, Yung-Chun Hsieh, Yi-Kai Su, Chun-Yi Lin, Shun-Mao Yang, Bor-Ran Li, Yang-Hsiang Chan

**Affiliations:** † Department of Applied Chemistry, National Yang Ming Chiao Tung University, Hsinchu 30010, Taiwan; ‡ Department of Surgery, National Taiwan University Hospital, Hsinchu Branch, Hsinchu 30010, Taiwan; § Institute of Biomedical Engineering, National Yang Ming Chiao Tung University, Hsinchu 30010, Taiwan; ∥ Center for Emergent Functional Matter Science, National Yang Ming Chiao Tung University, Hsinchu 30010, Taiwan; ⊥ Department of Medicinal and Applied Chemistry, Kaohsiung Medical University, Kaohsiung 80708, Taiwan

## Abstract

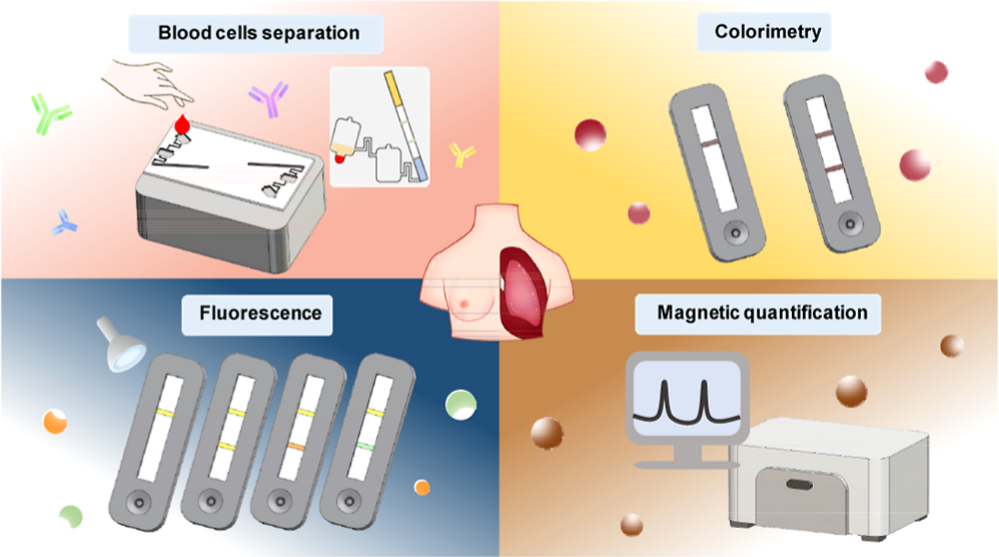

During the COVID-19 pandemic, the use of lateral flow assays (LFAs) expanded significantly, offering testing beyond traditional health care. Their appeal lies in the ease of use, affordability, and quick results. However, LFAs often have lower sensitivity and specificity compared with ELISA and PCR tests. Efforts to improve LFAs have increased detection times and complexity, limiting their use in large-scale point-of-care settings. To address this, we propose a novel approach using probes that generate multiple signals to enhance the sensitivity and selectivity. This concept also allows multiplexed LFAs to detect multiple analytes concurrently. We developed a trimodal probe that integrates fluorescence, color, and magnetism into a single nanohybrid. The strong plasmonic absorption and high fluorescence of Au nanoparticles and polymer dots enable qualitative and semiquantitative diagnosis, while the magnetic signal facilitates accurate quantitative measurements. As proof-of-concept targets, we selected CYFRA 21-1 and CA15-3, biomarkers for lung and breast cancer, respectively. This trimodal LFA demonstrated a remarkable detection limit of 0.26 ng/mL for CYFRA 21-1 and 2.8 U/mL for CA15-3. To the best of our knowledge, this is the first platform of a trimodal LFA with multiplexing ability. The platform’s accuracy and reliability were validated using clinical serum samples, showing excellent consistency with electrochemiluminescence immunoassay results. This universal concept can be applied to other targets, paving the way for the next-generation LFAs.

## Introduction

In 2003, the World Health Organization Special Programme for Research and Training in Tropical Diseases (WHO/TDR) established a set of standards for an optimal diagnostic test applicable across all tiers of health care in developing regions.^1^ These guidelines, defined as the ASSURED criteria (affordable, sensitive, specific, user-friendly, rapid, equipment-free, delivered), serve as the gold standard for point-of-care (POC) testing for infectious tropical diseases and sexually transmitted infections.^2−4^ Over the last 20 years, a range of POC diagnostic platforms have emerged to meet these requirements. Among them, lateral flow assays (LFAs) have garnered substantial recognition for their affordability and simplicity, particularly in detecting serum biomarkers, viruses, toxins, bacteria, and proteins.^5−7^ The significance of LFAs became increasingly evident in late 2019 during intense scientific efforts to combat the rapidly spreading SARS-CoV-2 virus. Nonetheless, the effectiveness of LFA is constrained by its limited sensitivity and specificity compared to traditional laboratory tests. Moreover, there is an urgent demand to create multiplexed LFAs capable of detecting and quantifying multiple analytes simultaneously on a single test strip. This necessity arises due to reasons of reduced cost, decreased sample volume, and prompt discrimination among disorders with similar symptoms, all while adhering to ASSURED criteria. However, several challenges persist in achieving this goal, such as potential cross-reactivity among affinity proteins targeting different analytes, physical constraints of multiplexed test areas on an LFA strip, and the complexity involved in clinical validation.^8,9^

Since the 2010s, lung cancer has maintained its position as the primary cause of cancer-related fatalities globally. Around 12.4% of newly reported cancer cases worldwide in 2022 were attributed to lung cancer, with a mortality rate of 18.7%, marking it as the most prevalent and deadly type of cancer.^10^ In 2022, breast cancer stood as the second most common cancer globally, comprising around 11.5% of all cancer cases, with approximately 2.3 million new diagnoses.^10^ Despite advancements, it continues to be the fifth most common cause of cancer-related mortality globally, claiming about 666,103 lives. Among women, breast cancer represents a significant burden, contributing to 23.8% of all cancer incidences and 15.4% of cancer-associated fatalities, according to the Globocan 2022 database as of August 2, 2024.^10^ In other words, 1 out of every 4 cancer patients will be diagnosed with either breast or lung cancer in 2022. Despite significant advancements in diagnostic methods like MRI, CT scans, and PET scans, along with a variety of treatments including surgery, radiotherapy, and chemotherapy, the latest statistics from the United Kingdom and United States show that the 5 year age-standardized survival rate for breast cancer is over 80%.^5^ In contrast, lung cancer exhibits a significantly lower 5 year survival rate, below 20%.^5^ In 2016, nonsmall-cell lung cancer (NSCLC) represented 78.06% of lung cancer cases in males and 91.86% in females in Taiwan.^11^ Upon tumor diagnosis, prognostic biomarkers offer insights into the disease’s potential trajectory, including recurrence, progression, and overall patient survival, independent of treatment.^12,13^ These markers may also reflect tumor burden, aiding in cancer staging, such as the tumor-node-metastasis classification. Common examples include carcinoembryonic antigen (CEA) for colorectal cancer, carbohydrate antigen 19-9 (CA19-9) for pancreatic cancer, cytokeratin 19 fragment (CYFRA21-1) for NSCLC, and carbohydrate antigen 15-3 (CA15-3) for breast cancer.^14,15^ The information derived from these biomarkers aids clinicians in determining whether aggressive or extended treatment strategies are warranted. Additionally, certain prognostic biomarkers have been employed to predict the efficacy of chemotherapy because it is preferable in a clinical setting and precision medicine.^16^ Therefore, there is a pressing need for a rapid, sensitive multiplex platform for simultaneous detection of NSCLC (CYFRA21-1) and breast cancer (CA15-3), facilitating improved clinical decision-making.

Aiming at designing a new type of LFA with multiplexed ability, we herein create the first example of a trimodal LFA with enhanced diagnostic sensitivity and specificity for multiplexing readout. Specifically, we take advantage of the ultrahigh fluorescence of semiconducting polymer dots (Pdots) and integrate them with Au nanoparticles and MnFe_2_O_4_ magnetic nanoparticles (MNPs) as the reporter to realize a triple-mode LFA. The intense emission intensity emitted by Pdots^17−35^ ensures superior sensitivity, surpassing traditional LFAs by 1–2 orders of magnitude.^36−38^ Importantly, the strong surface plasmonic absorption of Au nanoparticles enables the prompt interpretation of qualitative results by the naked eyes. The multicolor fluorescence signals of Pdots facilitate simultaneous detection of multiple analytes, while the magnetic signals from MNPs allow for quantitative measurements with minimal interference from background signals in the blood matrix. Furthermore, we developed a portable microfluidic device to complement the test strip. This innovation allows for the rapid separation of plasma from a single drop of whole blood on-site, which is then directly introduced into the sample pool of the test strip. As a proof-of-concept, we applied two prognostic biomarkers, CYFRA21-1 and CA15-3, from clinical serum samples of NSCLC and breast cancer patients, respectively, in this trimodal LFA. The results obtained from this approach demonstrated good agreement with those measured by ELISA, demonstrating the accuracy and reliability of this LFA platform. By coupling the microfluidic chip with the trimodal LFA, we truly accomplish POC objectives for timely tumor biomarker detection from one drop of whole blood, at home or in the field, and this approach can be readily adapted for other target analytes. This novel trimodal LFA opens up new possibilities for rapid and precise POC cancer diagnostics.

## Experimental Section

### Fabrication of the Portable Microfluidic Centrifugal Device^39^

The outer shell of the centrifugal device was manufactured using polylactic acid materials through 3D printing (Zortrax M200, QTS Co., Ltd., Taiwan), while the centrifugal platform was constructed using photopolymer resin using 3D printing (Sonic Mini 8K, Phrozen, Taiwan). Then, a 12 V brush DC geared motor (GA25-370, Centenary Materials Co., Ltd., Taiwan) was integrated into the device and powered to facilitate its operation. The microfluidic chip was fabricated using poly(methyl methacrylate) as the substrate material and cut by a CO_2_ laser (MK430, 3-Axle Technology Co., Ltd., Taiwan). Subsequently, we used a scanning and cutting machine (SDX1200, Brother Ltd., Taiwan) to punch the inlet and outlet in the polyethylene terephthalate film (3 M 89605, Ming Hong Material Co., Ltd., Taiwan), which were then adhered to both the upper and lower surfaces of the channels to form a microfluidic channel.

### Preparation of Au Nanoparticles^40^

255 mL portion of HAuCl_4_ (1 mM) was transferred to a 500 mL two-neck flask and heated to boiling at 130 °C. Following this, 42.5 mL of sodium citrate (38.8 mM) was rapidly added, and the mixture was stirred for 10 min. Subsequently, the solution was stirred at room temperature for another 15 min. Au nanoparticles with an absorption wavelength of approximately 520 nm were obtained and can be stored in the refrigerator for months.

### Preparation of MnFe_2_O_4_ Magnetic Nanoparticles^41^

360 mg of FeCl_3_·6H_2_O, 132 mg of MnCl_2_·4H_2_O, 10 mL of ethylene glycol, and 10 mL of diethylene glycol were sequentially introduced into a 250 mL round-bottom flask. The solution was then heated and stirred at 120 °C. After complete dissolution of FeCl_3_·6H_2_O and MnCl_2_·4H_2_O, 2.0 g of polyvinylpyrrolidone was added, and the mixture was stirred for 10 min; then heating was stopped. Next, 1.50 g of sodium acetate was incorporated, and the mixture was stirred for 0.5 h. The solution was then placed into a Teflon-lined hydrothermal autoclave and heated in an oven at 180 °C for 10 h. After the reaction was complete, the product was rinsed five times with deionized H_2_O to eliminate impurities and then placed into vacuum at 65 °C for 4 h to obtain MnFe_2_O_4_ MNPs.

### Surface Functionalization of MNPs^42^

For the preparation of MNPs coated with TC6FQ Pdots, first, a solution of 0.25 mg/mL MNPs and 1 mg/mL of branched polyethylenimine (PEI, *M*_W_ = 25,000) in deionized water was prepared, and then 1 mL of the magnetic nanoparticle solution was added with 250 μL of PEI under ultrasonic agitation for 30 min. MNPs were separated from the liquid by using magnetic separation to remove an excessive amount of PEI. The MNPs were rinsed three times by deionized H_2_O and finally resuspended in 1 mL of deionized H_2_O for further use. For the fabrication of MNPs coated with PFCN Pdots, 200 μL of PEI was used instead of 250 μL.

### Preparation of Pdots^38^

TC6FQ, PFCN, carboxymethyl-PEG-DSPE (CM-DSPE, *M*_W_ = 2000), and cumene-terminated polymer containing 75% styrene (PSMA, Mn ∼1900) were separately dissolved in THF with a concentration of 1 mg/mL (1000 ppm). In a 20 mL sample bottle, 2.5 mL of THF, 100 μL of TC6FQ or PFCN, 15 μL of CM-DSPE, and 5 or 10 μL of PSMA were sequentially added, and then the bottle was shaken to mix the contents evenly into a THF mixture. In another 20 mL sample bottle, 5 mL of deionized H_2_O was placed under ultrasonic agitation, and the above THF mixture was quickly injected during the agitation. The as-prepared solution was heated under nitrogen at 70 °C for about 20 min until the THF completely evaporated, confirming that the remaining aqueous solution is approximately 4 mL. The Pdot solution was then cooled to ambient temperature. The as-prepared Pdot solution was filtered by using a 0.22 μm cellulose acetate membrane syringe filter to eliminate larger particles or aggregates.

### Fabrication of MNP@AuNP@PFTC6FQ and MNP@AuNP@PFCN Nanohybrid Probes

For the capping of Pdots onto the surface of MNPs, 2 mL of PFTC6FQ or PFCN Pdot solution was first mixed with 150–250 μL of Au nanoparticles. After that, 1 mL of PEI-fabricated MNPs was added into the mixture under sonication for 20 min. Magnetic separation was exerted to separate the MNPs from the liquid, removing excess Pdots and gold nanoparticles. The MNPs were then washed three times with deionized H_2_O and finally resuspended in 1 mL of deionized H_2_O.

### Surface Modification of MNP@AuNP@Pdot Nanohybrids

1 mL portion of sodium citrate (2 mg/mL in water) was added into 1 mL of MNP@Au@Pdot solution under sonication for 15 min. Subsequently, magnetic separation was used to separate the MNP@AuNP@Pdot nanohybrids from the liquid, removing excess sodium citrate. The MNP pellet was then washed three times with deionized water and finally resuspended in 1 mL of deionized water.

### Antibody Conjugation of MNP@AuNP@PFTC6FQ and MNP@AuNP@PFCN

To functionalize CYFRA21-1 antibodies onto the surface of MNP@AuNP@PFTC6FQ, a vial was prepared by adding 1 mL of MNP@AuNP@PFTC6FQ, 20 μL of 5% (w/w) PEG (Mn = 3350), 20 μL of 1 M HEPES buffer, 20 μL of freshly prepared 1-ethyl-3-(3-(dimethylamino)propyl)carbodiimide (EDC, 5 μg/mL), 5 μL of *n*-hydroxysuccinimide (NHS, 5 μg/mL), and 7 μL of CYFRA21-1 antibodies (1 mg/mL, product code: 10-2461). The reaction mixture was then stirred at room temperature for 4 h. Following this, magnetic separation was used to remove the supernatant. The obtained pellet was rinsed with deionized H_2_O for three times and finally resuspended in 200 μL of pure water, resulting in the formation of CYFRA21-1-conjugated MNP@AuNP@PFTC6FQ nanohybrids. For the preparation of CA15-3-conjugated MNP@AuNP@PFCN nanohybrids, PFCN and CA15-3 (product code: 136549) antibodies were used in place of PFTC6FQ and CYFRA21-1 antibodies, respectively.

### Operation of the Portable Microfluidic Centrifugal Device Integrated with a Lateral Flow Strip

(1) The polyethylene terephthalate film was applied to the lower layer of the microfluidic chip and placed on the assembled test paper. (2) the film was applied to the upper layer of the microfluidic chip and placed on the centrifuge. (3) The third stage of the microfluidic chip was sealed with tape. (4) Fingertip blood was mixed with 40 μL of PBS, and the mixture was added to the first stage of the microfluidic chip. (5) The first and second stages of the microfluidic chip were sealed with tape. (6) The centrifuge was turned on; centrifugation was carried out for 30 min to separate blood cells from serum. (7) The tape was torn off from the second and third stages of the microfluidic chip, the prepared reagent was added to the second stage, and then the third stage was sealed with tape. (8) The centrifuge was turned on, centrifugation was carried out for about 10 s to allow the reagent added in the second stage to settle, and then the tape was torn off from the first stage. (9) The centrifuge was turned on, centrifugation was carried out for 1 min to allow the serum centrifuged in the first stage to flow into the second stage for mixing, and then the tape was torn off from the third stage. (10) The centrifuge was turned on, and centrifugation was carried out for 3 min to allow all mixed liquids to flow onto the sample pad of the third stage test paper, with a waiting time of 15 min to interpret the results. The complete process can be viewed in the demo video (Video S1).

## Results and Discussion

Aiming to create a trimodal LFA capable of colorimetric, fluorescent, and magnetic readouts for the detection of CYFRA21-1 and CA15-3 at the same time for patient whole-blood samples, a hybrid nanomaterial with three distinct signals was engineered. Herein, MNPs were employed as the core and then coated with Au nanoparticles, followed by the capping of fluorescent Pdots onto the outermost layer of the nanoparticle surface. Ideally, the magnetic property acts as both the quantitative reporter and the tool for probe purification. Meanwhile, Au nanoparticles serve as qualitative reporters, easily recognizable by the naked eye. Multicolor fluorescent Pdots are well-suited for multiplexed detection, allowing for the monitoring of color differences on the test line to detect CYFRA21-1/CA15-3 individually or simultaneously.

### Design of the Portable Microfluidic Centrifugal Device

For LFA platform using whole blood samples without pretreatment, several challenges have been encountered:^38,43^ (1) At least 3 mL of whole blood is required for immunological analysis; and (2) red blood cells interfere with the colorimetric reaction of the strip upon introduction of whole blood. To address these issues, facilitating the removal of red blood cells from small volumes of whole blood to extract plasma and apply it to the lateral flow strip has been proposed herein. Traditional strategies involve in adding a filter to the middle of the sample pad to filter out red blood cells or using centrifugation in capillaries to separate plasma.^44^ However, using a filter membrane in the lateral flow strip would limit the adsorption or aggregation of nanoparticle-based probes inside the membrane, which greatly affects the reproducibility of detection. On the other hand, centrifugation in capillaries is still hindered by the higher hematocrit of whole blood, requiring a lengthy process for complete cell separation with a smaller sample yield. Moreover, breaking the capillary is necessary to drop the detection reagent or strip, which remains uneconomical and impractical.^45^ Therefore, a single-step method for plasma separation compatible with nanoprobes for lateral flow strip detection is in high demand.

**Figure 1:**
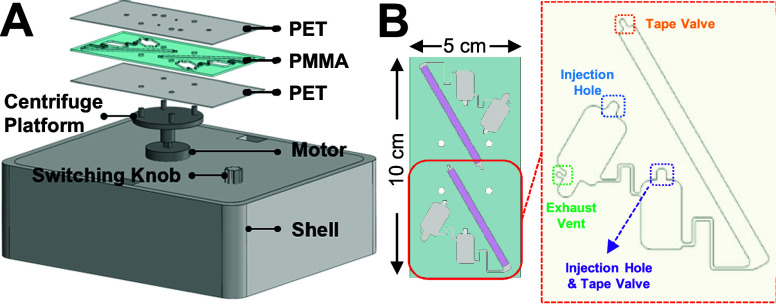
(A) Design of the portable microfluidic centrifugal device for blood separation. (B) Enlarged view of the disposable PMMA microfluidic chip that can be used twice (up and down) for a single chip.

To address the aforementioned issues, here we developed an integrated microfluidic centrifugal device that combines centrifugation with a lateral flow strip (Figure 1A). Through adjustments in the rotational speed and pressure control of the tape valve, only a single drop of whole blood (∼10 μL) is required to undergo single-step centrifugation with running buffer and probe mixing (Figure 1B). This design can effectively reduce the hematocrit levels in blood and enable the on-site operation. Therefore, this portable microfluidic centrifugal device eliminates unnecessary steps and time as compared to conventional large volume blood centrifugation devices. Moreover, the platform can conduct tests within sealed channels to eliminate the possibility of blood contamination. The combination of the portable microfluidic centrifugal device with the test strip demonstrates the enormous potential of this device in the future development of POC.

### Preparation of MNP@AuNP@Pdot Hybrid Nanomaterials

**Scheme 1:**
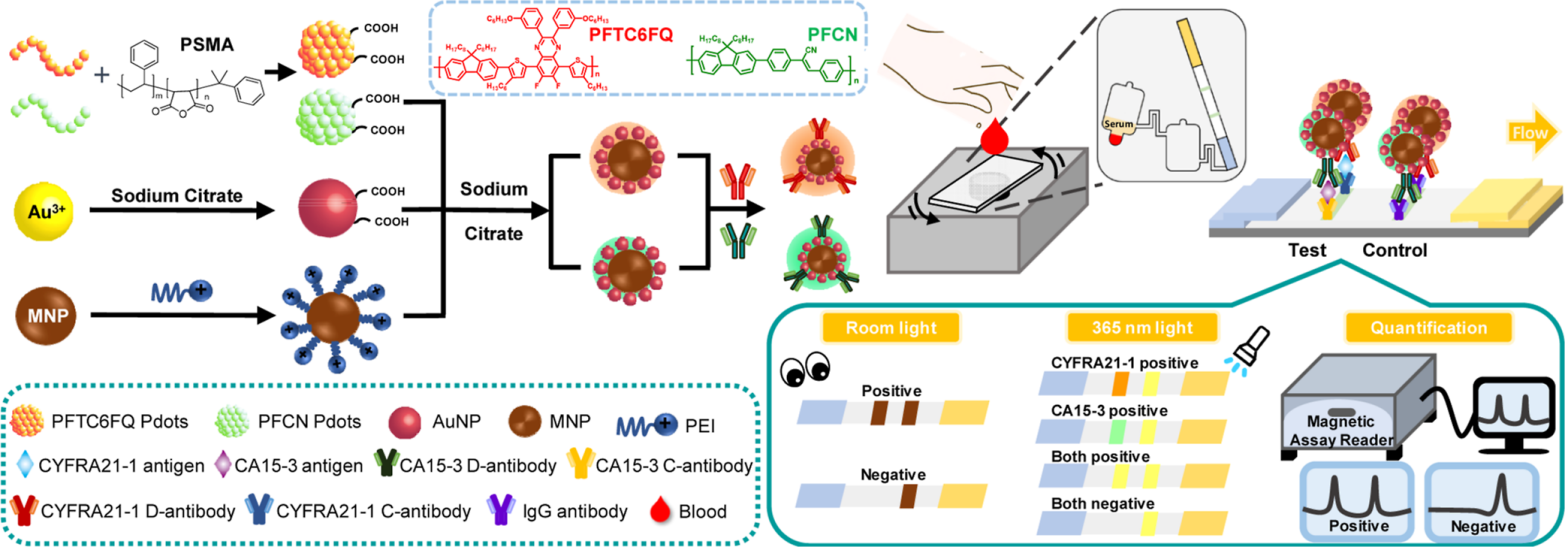
Schematic Depicting the Design of MNP@Au@Pdot-Based Lateral Flow Test Strip for Multiplexed Detection First, semiconducting polymers PFTC6FQ and PFCN were fabricated as Pdots through nanoprecipitation. In a separate step, citrate-coated Au NPs with negative charges were synthesized. Additionally, PEI-coated MNPs with positive charges were prepared. The Au NPs were then mixed with PFTC6FQ or PFCN Pdots, followed by the addition of MNPs to create MNP@AuNP@Pdot nanohybrids. These nanohybrids were conjugated with detection antibodies for CYFRA21-1 or CA15-3. A portable microfluidic centrifugal device was used to separate plasma from blood, which was then loaded into the trimodal lateral flow test strip. The test strip had a test line fabricated with capture antibodies for CYFRA21-1, CA15-3, or both for multiplexed detection, with a control line fabricated with the IgG secondary antibody. The presence of target antigens could be visually detected by the color shade or fluorescence signal of the test zone. For quantitative measurement, the magnetic signals on the control and test lines were measured using a magnetic assay reader to accurately determine the concentration of the target antigens.

**Figure 2:**
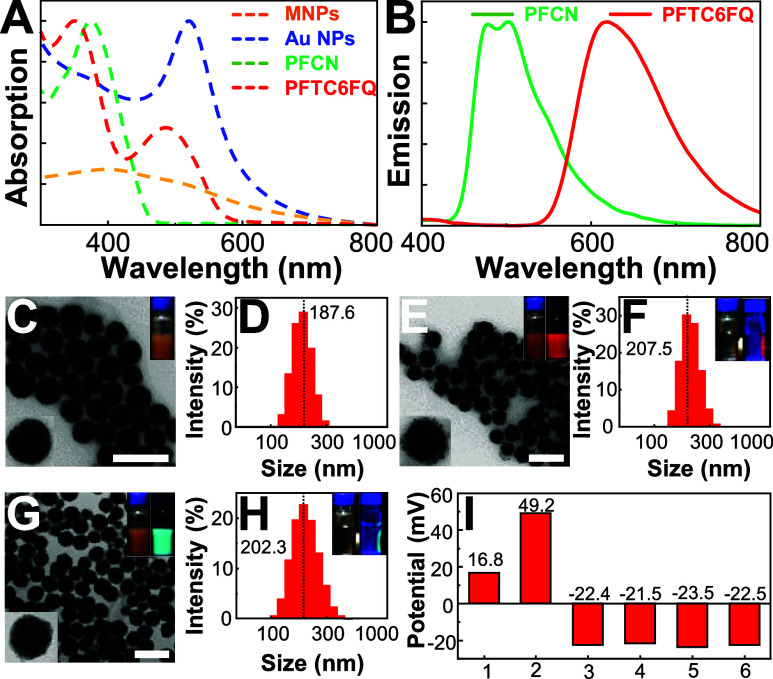
(A) Absorption spectra in aqueous solutions for MNPs, Au NPs, PFCN, and PFTC6FQ Pdots. (B) Emission spectra in aqueous solutions for MNP@AuNP@PFCN and MNP@AuNP@PFTC6FQ Pdots. (C) TEM image of MNPs and (D) their corresponding hydrodynamic diameters obtained by DLS. The inset in (C) represents the photograph of MNPs. (E) TEM image of MNP@AuNP@PFTC6FQ nanohybrids and (F) their corresponding hydrodynamic diameters obtained by DLS. The inset in (E) exhibits the images of MNP@AuNP@PFTC6FQ under room (left) and 365 nm (right) light. The inset in (F) shows the photographs of MNP@AuNP@PFTC6FQ with a magnet on the vial wall under room (left) and 365 nm (right) light, presenting the fluorescent and magnetic properties these nanohybrids. (G) TEM image of MNP@AuNP@PFCN nanohybrids and (H) their corresponding hydrodynamic diameters obtained by DLS. The inset in (G) displays the images of MNP@AuNP@PFCN under room (left) and 365 nm (right) light. The inset in (H) shows the photographs of MNP@AuNP@PFCN with a magnet on the vial wall under room (left) and 365 nm (right) light. (I) Zeta potentials of MNPs (no. 1), PEI-functionalized MNPs (no. 2), MNP@AuNP@PFTC6FQ (no. 3), citrate-fabricated MNP@AuNP@PFTC6FQ (no. 4), MNP@AuNP@PFCN (no. 5), and citrate-fabricated MNP@AuNP@PFCN (no. 6).

To endow probes with trimodal signals as illustrated in Scheme 1, we first synthesized MnFe_2_O_4_ MNPs with a mean hydrodynamic diameter of 187.6 nm (Figure 2C). The as-prepared positively charged MNPs (ζ = +16.8 mV) were then coated with branched PEI to increase the zeta potential to +49.2 mV (Figure 2I). On the other hand, negatively charged Au NPs with an absorption maximum of 520 nm and multicolor emissive Pdots, including PFCN and PFTC6FQ Pdots, were prepared separately. Subsequently, the cocoating of Au NPs and Pdots onto the surface of MNPs yielded MNP@AuNP@Pdot nanohybrids with average diameters of 207.5 and 202.3 nm for MNP@AuNP@PFTC6FQ and MNP@AuNP@PFCN, respectively. The enlarged TEM images of the nanohybrids in Figure 2E–G demonstrate the successful encapsulation of Au NPs and polymers on the surface of MNPs. The resulting nanohybrids exhibit optical and magnetic properties, embedding colorimetric, fluorescent, and magnetic triple-readout signals into a single probe. It is important to note that the ratios of Au NPs to Pdots are carefully controlled to preserve the visible color of the Au NPs while avoiding emission quenching behaviors of the Pdots due to electron or energy transfer from the Au nanoparticles.^46^ The negatively charged surface of MNP@AuNP@Pdot nanohybrids is advantageous for negatively charged nitrocellulose membranes of test strips, effectively preventing nonspecific adsorption through electrostatic interactions.

### Detection Principle of Trimodal MNP@AuNP@Pdot-Based LFA

Following the synthesis, the CYFRA21-1 detection antibody was conjugated with the resulting MNP@AuNP@PFTC6FQ nanohybrids, while the CA15-3 antibody was attached to the MNP@AuNP@PFCN nanomaterials. This conjugation endowed the MNP@AuNP@Pdot nanohybrids with both a trimodal readout and specific targeting capabilities. A control zone along a test zone were established on LFA strip. The control line contained unmodified IgG secondary antibodies, while the test zone was equipped with CYFRA21-1 capture antibodies, CA15-3 capture antibodies, or both for multiplexing detection. The assembly included the sample pad, reaction membrane, and absorbent pad, all enclosed within a plastic casing. Whole blood samples (20–40 μL) underwent microfluidic-centrifugation-assisted precipitation to obtain blood plasma (refer to Video S1). The plasma was combined with the running buffer and introduced into the sample pad of the test strip. Capillary force caused the buffer and serum with analytes to move along the strip from the sample pad to the absorbent pad. When only CYFRA21-1 was present, MNP@AuNP@PFTC6FQ hybrids were attached on the test zone, showing a distinct red color from the Au NPs visible to the naked eye along with strong red emission from PFTC6FQ with UV excitation. Simultaneously, the magnetic signals on the test line could be quantified. In the presence of only CA15-3, MNP@AuNP@PFCN hybrids were captured on the test line, leading to a vivid red color from Au NPs and a green emission of PFCN. The test line’s color and fluorescence changed according to their concentrations. The control line consistently appeared dark red, while the test line’s fluorescence ranged from yellow-white to white, contingent upon the ratio of PFTC6FQ/PFCN. The test line color enabled quick identification of CYFRA21-1, CA15-3, or both. Additionally, magnetic intensity ratios between the test and control lines (T/C) allowed for the quantitative measurement of CYFRA21--1 and CA15-3 levels. This fluorescent, colorimetric, and magnetic trimodal signal readout LFA is poised to serve as an exemplary model for the multiplexed diagnosis of cancer biomarkers.

**Figure 3:**
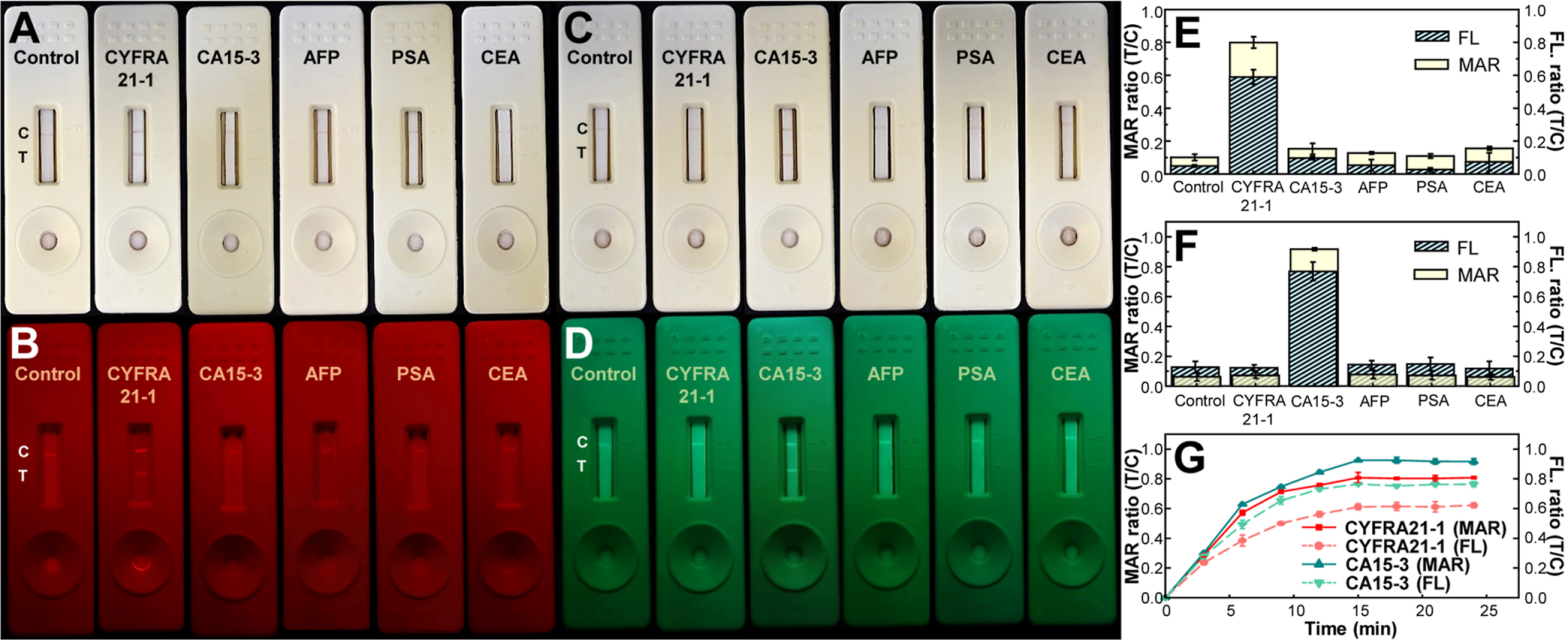
Evaluation of selectivity with MNP@AuNP@PFTC6FQ and MNP@AuNP@PFCN nanohybrids targeting diverse antigens. (A) Images of test strips with MNP@AuNP@PFTC6FQ nanohybrids under room light. (B) Corresponding fluorescent images with 365 nm light irradiation with a 675 ± 75 nm band-pass filter after exposure to analytes with various antigens (10 ng/mL for CYFRA21-1, AFP, PSA, and CEA; 100 U/mL for CA15-3). (C) Test strip images with MNP@AuNP@PFCN nanohybrids under room light. (D) Corresponding fluorescent images under 365 nm light with a 525 ± 25 nm band-pass filter after reaction with analytes containing various antigens (10 ng/mL for CYFRA21-1, AFP, PSA, and CEA; 100 U/mL for CA15-3). (E) Ratios of fluorescence (slash column) and magnetism (yellow column) intensities of T/C measured in (A,B). (F) Ratios of fluorescence (slash column) and magnetism (yellow column) intensities of T/C measured in (C,D). (G) Fluorescent (dashed lines) and magnetic (solid lines) T/C ratios over various reaction times for CYFRA21-1 using MNP@AuNP@PFTC6FQ probes and for CA15-3 using MNP@AuNP@PFCN probes.

Assessing the specificity of MNP@AuNP@PFTC6FQ and MNP@AuNP@PFCN nanoprobes toward CYFRA21-1 and CA15-3 represents the initial phase in validating the practical utility of this trimodal LFA. Figure 3A illustrates the high specificity of MNP@AuNP@PFTC6FQ probes for CYFRA21-1, with ignorable nonspecific binding phenomenon for other antigens like CA15-3, AFP, PSA, and CEA. Enhanced distinguishability of fluorescence signals (Figure 3B) indicates ignorable signal of PFTC6FQ on the test zones, except in samples with CYFRA21-1, reaffirming the significant selectivity of MNP@AuNP@PFTC6FQ probes. In a similar scenario, MNP@AuNP@PFCN nanoprobes exhibit excellent specificity toward CA15-3 (Figure 3C,D). The emission signal ratio of the test zone to the control zone is over three times higher for MNP@AuNP@PFTC6FQ to CYFRA21-1 than for other reagents (Figure 3E). Conversely, magnetic signals of test to control line ratios exceed five times higher for MNP@AuNP@PFTC6FQ to CYFRA21-1 than for other analytes, a phenomenon also observed for MNP@AuNP@PFCN (Figure 3F). These findings suggest that magnetic signals offer superior sensitivity to fluorescence signals, owing to neglectable magnetic background interference from serum and nitrocellulose membranes. Leveraging color/fluorescence intensities for qualitative interpretation and magnetic signals for precise quantitative detection is achievable in this trimodal LFA platform. Additionally, the platform shows high specificity for CYFRA21-1 and CA15-3, minimizing both false negatives and false positives. Rapid test result acquisition is essential for on-site patient care. Analysis of emission intensities on the test/control zones at various running periods (Figure 3G) reveals that a 15 min reaction period is sufficient for accurate interpretation with both probes.

### Qualitation and Quantitation of CYFRA21-1 and CA15-3 Serum Samples

**Figure 4:**
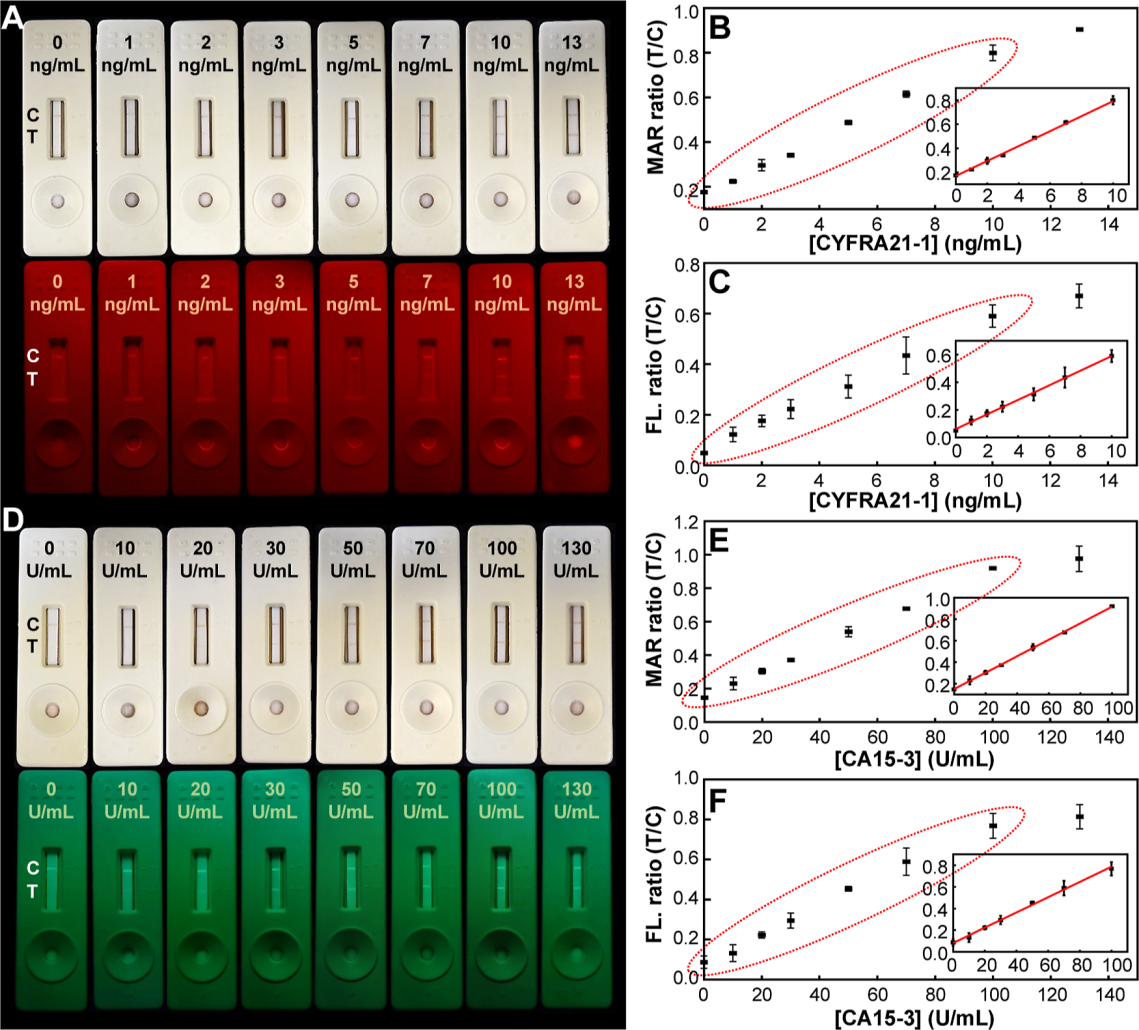
Quantitative performance of CYFRA21-1 and CA15-3 with MNP@AuNP@PFTC6FQ and MNP@AuNP@PFCN nanohybrids. (A) Images of test strips utilizing MNP@AuNP@PFTC6FQ nanoprobes under room light (upper panel) and their respective emission images under 365 nm light excitation (bottom panel) with a band-pass filter of 675 ± 75 nm after exposure to analytes containing CYFRA21-1 concentrations ranging from 0 to 13 ng/mL. The detection dynamic ranges of CYFRA21-1 based on (B) magnetic signals and (C) fluorescence signals. (D) Test strip photographs using MNP@AuNP@PFCN nanoprobes under room light (upper panel) and their respective emission images under 365 nm light excitation (bottom panel) with a band-pass filter of 525 ± 25 nm after exposure to analytes containing CA15-3 concentrations ranging from 0 to 130 U/mL. Corresponding detection dynamic ranges of CA15-3 based on (E) magnetic signals and (F) fluorescence signals.

In lateral flow test strips, rapid screening and accurate determination of the target concentration are equally crucial. When detecting CYFRA21-1 using MNP@AuNP@PFTC6FQ probes (Figure 4A, upper panel), the test lines become visible to the naked eye when CYFRA21-1 concentrations exceed 3 ng/mL. A cutoff value is set to ensure that 95% of healthy individuals test negative, achieving a selectivity of 95%. For CYFRA21-1, a concentration above 3.3 ng/mL signifies a positive detection of squamous cell carcinoma.^47^ Therefore, rapid colorimetric interpretation facilitates immediate screening to ascertain whether the CYFRA21-1 cutoff value is reached. Under UV illumination (Figure 4A, bottom panel), the fluorescence brightness gradually increases with rising CYRFA21-1 concentrations. Moreover, fluorescence signals offer a more sensitive determination of the cutoff value compared to the colorimetric method, suggesting a promising semiquantitative approach. On the other hand, based on magnetic signals (Figure 4B), the detection sensitivity surpasses that of fluorescence signals (Figure 4C). These findings suggest that colorimetric, fluorescent, and magnetic trimodal signals serve distinct roles in qualitative, semiquantitative, and quantitative reporting, respectively. A similar scenario is observed for CA15-3 concentrations exceeding 20 U/mL (Figure 4D), prompting doctors to recommend further evaluations. This capability allows for rapid interpretation of test results, making it ideal for fast, large-scale qualitative diagnosis of biomarker thresholds under clinical environments.

Sensitivity is primarily assessed through the detection limit and the quantitative linear range. Using magnetic signals (Figure 4B), MNP@AuNP@PFTC6FQ probes exhibited a detection range for CYFRA21-1 from 0 to 10 ng/mL, closely matching the CYFRA21-1 cutoff value of 3.3 ng/mL. Likewise, MNP@AuNP@PFCN probes showed a broad dynamic range from 0 to 100 U/mL for CA15-3 (Figure 4E). The limits of detection for CYFRA21-1 and CA15-3 were 0.26 ng/mL and 2.8 U/mL, respectively.

### Multiplexed Diagnosis of CYFRA21-1/CA15-3 in LFA

**Figure 5:**
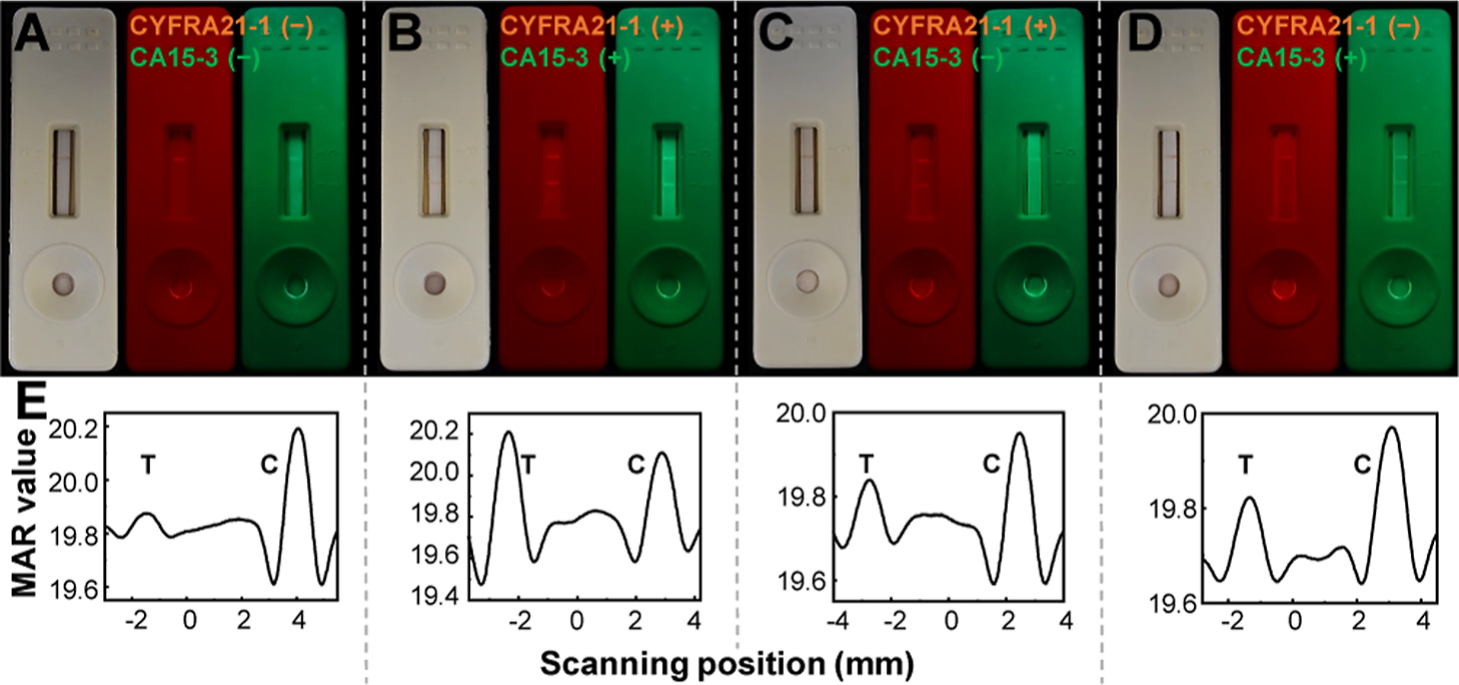
Multiplexing evaluation for detecting CYFRA21-1 and CA15-3 in serum analytes. Test strip images under room light (left), 365 nm light with a 675 ± 75 nm band-pass filter (middle), and 365 nm light with a 525 ± 25 nm band-pass filter (right) for analytes with CYFRA21-1 and CA15-3 at (A) 0 ng/mL and 0 U/mL, (B) 10 ng/mL and 100 U/mL, (C) 10 ng/mL and 0 U/mL, and (D) 0 ng/mL and 100 U/mL. (E) Corresponding magnetic signal measurements.

Pdots offer a distinct advantage in multiplexed detection due to their ability to emit multiple colors and their wide absorption spectra, in contrast to Au or MnFe_2_O_4_ NPs, which emit monochromatic signals. As illustrated in Scheme 1, the test line was functionalized with a combination of CYFRA21-1 and CA15-3 capture antibodies as a proof-of-concept. Magnetic signals can quantify the total levels of CYFRA21-1 and CA15-3, while fluorescence can differentiate between the two biomarkers. The effectiveness of this multiplexed detection is demonstrated in Figure 5. Simulated real analytes were prepared by adding serum samples with 10 ng/mL CYFRA21-1, 100 U/mL CA15-3, or both. The negative control, which did not contain CYFRA21-1 or CA15-3, exhibited negligible signal in the test line (Figure 5A), demonstrating minimal false-positive occurrences for this LFA system. In the presence of both CYFRA21-1 and CA15-3, two fluorescent test lines were observed under UV illumination with band-pass filters of 675 ± 75 and 525 ± 25 nm, respectively (Figure 5B), confirming multiplexing diagnosis of CYFRA21-1 and CA15-3. When only CYFRA21-1 was present, a distinct fluorescent test zone from MNP@AuNP@PFTC6FQ probes with negligible emission from MNP@AuNP@PFCN was observed (Figure 5C). When only CA15-3 was present, a noticeable green fluorescence was observed in the test tube (Figure 5D). These results demonstrate the high selectivity of these hybrid probes, enabling multiplexing detection in complex samples with minimal cross-interaction. However, magnetic intensity can be utilized for the quantitative calculation of the total amount of CYFRA21-1 and CA15-3 in serum analytes, although they cannot differentiate the presence of either CYFRA21-1 or CA15-3 alone.

### Quantitative Measurement of CYFRA21-1 and CA15-3 Levels in Clinical Blood Samples from Patients

**Figure 6:**
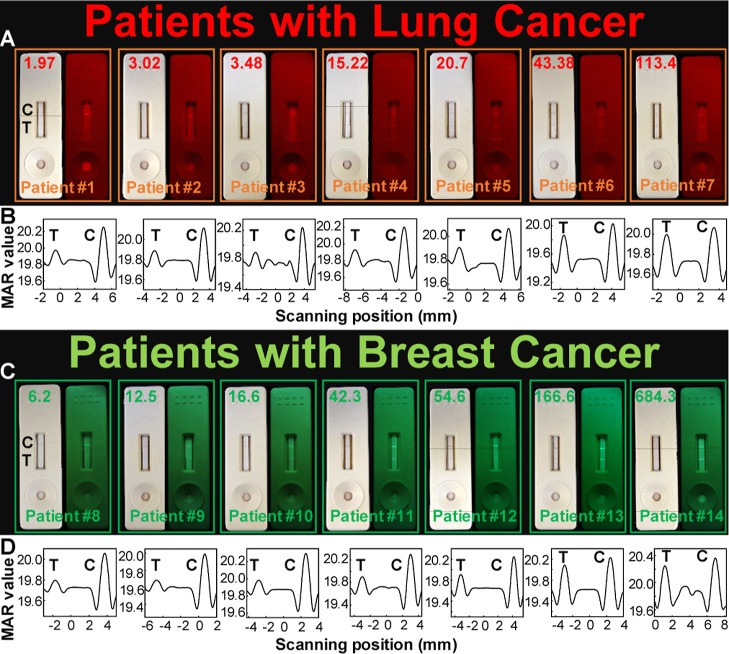
(A) Quantitative assessment of clinical samples from seven lung cancer patients at various stages using the MNP@AuNP@PFTC6FQ-based trimodal LFA. (B) Corresponding magnetic signals of control and test lines. (C) Quantitative evaluation of clinical samples from seven breast cancer patients at various stages using the MNP@AuNP@PFCN-based trimodal LFA. (D) Corresponding magnetic signals of control and test lines. The left sides of the photographs in (A,C) display the testing results under room light, and the right sides display their emission intensities under 365 nm light excitation. The values shown on top of the test strips correspond to concentrations acquired from electrochemiluminescence immunoassay (ECLIA). Red numbers in (A) indicate CYFRA21-1 levels (ng/mL), while green numbers in (C) represent CA15-3 levels (U/mL).

The quantitative performance of this MNP@AuNP@Pdot-based trimodal LFA was assessed in patients with lung or breast cancers at various stages (Figure 6). As depicted in Figure 6A, the test zones displayed clear coloration under white light (left panels) and distinct emission under 365 nm light excitation (right panels). The visible red color on the test lines allows for quick visual screening (refer to Video S2), while the fluorescence intensities correlate with CYFRA21-1 concentrations (red numbers on the left panels, as determined by ECLIA in the hospital and detailed in Table 1) across various stages of lung cancer. Patient samples were diluted 2–15 times to make sure that the resulting biomarker values were within the detection linear ranges. Quantitative measurement of CYFRA21-1 levels in these patients by this LFA relied on the magnetic signals of the control and test lines (Figure 6B). The CYFRA21-1 concentrations obtained by LFA closely matched the clinical results determined by ECLIA (Figure S1A), indicating the promising application of this MNP@AuNP@Pdot-based trimodal LFA in clinical settings. A similar trend was observed for clinical samples from patients with breast cancer (Figure 6C,D). The CA15-3 levels obtained aligned well with those from ECLIA (Figure S1B), further affirming the practical utility of this trimodal LFA in clinical samples.

**Table 1:** Values of CYFRA21-1 and CA15-3 of Clinic Samples Measured by ECLIA

	[CYFRA] (ng/mL)	TNM stage	histology
patient #1	1.97	T1b/N0/M1c	adenocarcinoma
patient #2	3.02	T2b/N0/M0	adenocarcinoma
patient #3	3.48	T1c/N0/M0	adenocarcinoma
patient #4	15.22	T0/N0/M1b	suspect colon cancer recurrence with lung metastasis
patient #5	20.7	T2/N0/M1a	adenocarcinoma
patient #6	43.38	T2/N2/M1a	adenocarcinoma
patient #7	113.4	T4/N2/M1c	adenocarcinoma

	[CA15-3] (U/mL)	TNM stage	histology
patient #8	6.2	T1b/N0/M0	invasive lobular carcinoma
patient #9	12.5	T2/N0/M0	invasive carcinoma of no special type
patient #10	16.6	T1c/N0/M0	invasive micropapillary carcinoma
patient #11	42.3	T2/N0/M0	invasive ductal carcinoma
patient #12	54.6	T4b/N*x*/M1	invasive ductal carcinoma
patient #13	166.6	T4a/N1/M0	invasive ductal carcinoma
patient #14	684.3	T4a/N2a/M0	invasive ductal carcinoma

## Conclusions

In essence, we have pioneered the development of a novel trimodal LFA platform based on MNP@AuNP@Pdot hybrids in conjunction with microfluidic centrifugal chips for the qualitative and quantitative assessment of CYFRA21-1 and CA15-3 from a single drop of whole blood. This new LFA system enables on-site testing, with results interpreted within 15 min. Importantly, it enables simultaneous detection of CYFRA21-1 and CA15-3, key prognostic markers for lung and breast cancer, respectively. Additionally, it covers the detection ranges required for CYFRA21-1 (3.3 ng/mL) and CA15-3 (25 U/mL) cutoff values.^48^ We foresee that this user-friendly yet reliable LFA can be easily extended to various cancers, aiding in treatment outcome prediction and serving as a tool for regular patient follow-up post-treatment.

## Supporting Information Available

Additional experimental procedures: materials, synthetic routes, and characterization (PDF)
Testing processes in real samples: full procedure (MP4)
Demo of clinical samples (MP4)
