# Supplementary material for: Trimodal Multiplexed Lateral Flow Test Strips Assisted with a Portable Microfluidic Centrifugation Device
Source: Anal Chem. 2024 Sep 14;96(38):15238–47. doi: 10.1021/acs.analchem.4c02432 (PMC11428082; doi:10.1021/acs.analchem.4c02432)
Supplement: Supplementary file 1 — ac4c02432_si_001.pdf [file ac4c02432_si_001.pdf]

Supporting Information

Trimodal Multiplexed Lateral Flow Test Strips Assisted with Portable Microfluidic Centrifugation Device

Man-Wen Wang,[a†] Zong-Min Chen,[a†] Yung-Chun Hsieh,[b†] Yi-Kai Su,[c] Chun-Yi Lin,[a] Shun-Mao Yang,[b] Bor-Ran Li,*[c] and Yang-Hsiang Chan*[ade]*

[†]Authors contributed equally to this work

[a]Department of Applied Chemistry, National Yang Ming Chiao Tung University, Hsinchu, Taiwan 30010

[b]Department of Surgery, National Taiwan University Hospital, Hsinchu Branch, Hsinchu, Taiwan 30010

[c]Institute of Biomedical Engineering, National Yang Ming Chiao Tung University, Hsinchu, Taiwan 30010

[d]Center for Emergent Functional Matter Science, National Yang Ming Chiao Tung University, Hsinchu, Taiwan 30010

[e]Department of Medicinal and Applied Chemistry, Kaohsiung Medical University, Kaohsiung, Taiwan

E-mail: Yang-Hsiang Chan (yhchan@nycu.edu.tw)

Supporting Information

Contents

Experimental Procedures S3
Materials. S3
Synthetic Procedures of Polymers S4
Characterization and Preparation of LFA S5
Figure S1 .... S8
Figure S2 .... S8
Figure S3 .... S9
Figure S4 .... S9
Figure S5 .... S9
Figure S6 .... S10
Figure S7 .... S10
Table S1 .... S11
Table S2 .... S11
References: S11

Supporting Information

Experimental Procedures

Materials.

The chemicals except for bio-related reagents were obtained from Sigma-Aldrich, Acros, TCI, and Thermo Fisher. Highly pure water (18.2 MΩ•cm) was used throughout our experiments. Polystyrene graft ethylene oxide functionalized with carboxyl acid group (PS-PEG-COOH, M_n = 6500, PDI=1.3) was obtained from Polymer Source (Quebec, Canada) without further purification. Semiconducting polymers **PFCN**,[1] and **PFTC6FQ**[2] were synthesized according to the reported literatures. CYFRA21-1 antibodies and antigens were acquired from Fitzgerald (MA, USA). CA15-3 antibodies/proteins were purchased from USBiological (MA, USA). Mouse anti-human CA15-3 antibodies (Cat. No.: 136541 and 136549) were engineered onto the test line and the Pdot surface, respectively. Mouse anti-human CYFRA21-1 (catalog number: 10-2461 and 10-2462) were conjugated onto the probe and the test line, respectively. AffiniPure goat antimouse IgG (H+L) secondary antibodies (AB_2338447) were acquired from Jackson ImmunoResearch (West Grove, PA) for further fabrication on the control line. CA15-3 antigen (C0050-27) and CYFRA21-1 antigen (30-AC69) were diluted into the desired concentrations by PBS buffer upon use. Nitrocellulose membranes (5 µm, CNPC), sample pads (GFB-R4), conjugate release matrices (PT-R5), and absorbent pads (AP080) were obtained from mdi Membrane Technologies and then cut into desired the dimension to assemble to test strips, and then inserted into 4 mm plastic cassettes.

Supporting Information

Scheme S1. Synthetic routes for the **PFCN** conjugated polymer.

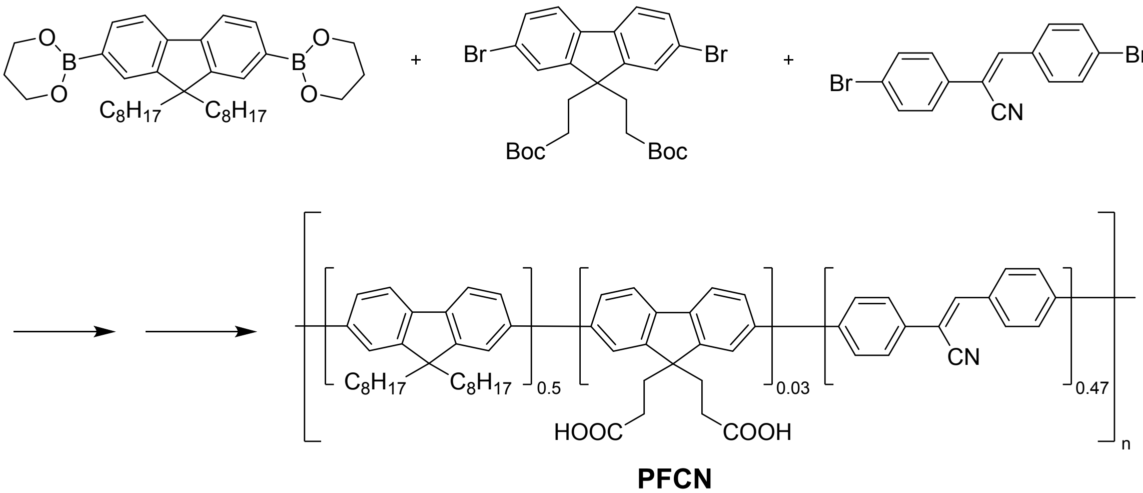

PFCN

Scheme S2. Synthetic routes for the **PFTC6FQ** conjugated polymer.

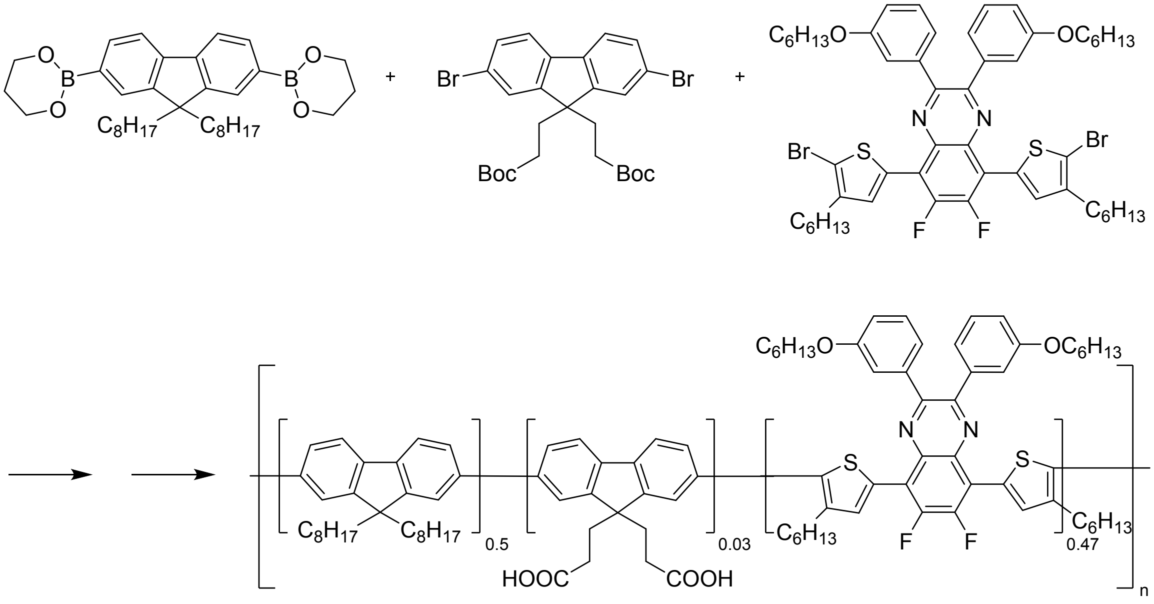

PFTC6FQ

Supporting Information

Characterization of MNP@AuNP@Pdot Nanohybrids

The UV/Visible spectra of the obtained nanohybrids were characterized by UV-visible spectroscopy (DB20S, Dynamica Limited). The average hydrodynamic radius was measured by the dynamic light scattering (DLS) instrument (Malvern Zetasizer Nano S) and TEM images of the as-prepared Pdots were measured by a Hitachi transmission electron microscope (HT7700) at an acceleration voltage of 100 kV. The preparation of TEM sample was prepared by carefully dropping 5 μL of Pdot aqueous solution (diluted by 6 times) was placed onto a carbon-coated grid and put into a dry cabinet for 24 h. The grid was further placed inside an oven at 80 °C for another 24 h to make sure water was completely evaporated. The fluorescence spectra were acquired on a FS5 spectrofluorometer (Edinburgh Instruments Ltd.).

Preparation of Test Strip by Using MNP@AuNP@Pdot-Antibody Probes

The assembly and the arrangement of different types of pads on the test strip was depicted in Scheme 1. First, the sample pad, and absorbent pad were all cut into a square size of 6 (length) x 3 (width) mm^2. The nitrocellulose membrane (3 mm in width) was fabricated with a test line composed of either CYFRA21-1 (10-2462, 2 mg/mL) or CA15-3 (136541, 0.5 mg/mL) antigens, and a control line composed of IgG secondary antibodies (0.45 mg/mL) by using an automated lateral flow reagent dispenser. For multiplexed detection, the test line was loaded with both CYFRA and CA15-3 detection antigens together. The test strip was placed under a vacuum environment for 3 min to make the water dry. After the fabrication of test and control lines, the test strip was assembled by integrating the absorbent pad with the sample pad on the nitrocellulose membrane. The test strip was then inserted into a 4-mm plastic cassette.

Detection of CA15-3/CYFRA21-1 by MNP@AuNP@Pdot-Based LFA

To prepare the running buffer, a mixture of various substances was combined. Specifically, 10 μL of 5% (w/w) Triton X-100, 3 μL of 1% (w/w) glycerol, 5 μL of 5% (w/w) polyethylene glycol 3000, 3 μL of 10% (w/w) BSA, and 3 μL of 5% (w/w) sucrose were mixed together. Additionally, 10 μL of MNP@AuNP@Pdot probes, X μL of the target

solution containing CYFRA21-1 and/or CA15-3, 5 μL of serum, and (61-X) μL of 20 mM HEPES buffer were added to the mixture. For the calibration samples used to establish the calibration curves, CYFRA21-1/CA15-3 were spiked into the running buffer. Volumes ranging from 0 to 20 μL of the spiked solution were added to the running buffer, resulting in a final volume of 0.1 mL. For the testing of whole blood samples, one-two drops of whole blood were mixed with 40 μL of PBS and then loaded into the microfluidic centrifugal chip. In the 7th step of the operation of microfluidic centrifugal device, 80 μL of running buffer was added. After that, the test strips were left undisturbed for a duration of 15 min until the results became visible. The fluorescence images of the test strips can be captured using a regular digital camera (e.g., Nikon Z7II) or a smartphone under a handheld 365 nm UV lamp (model: UVG-11, 4w, VWR International, LLC.). The camera setting is: 1) F-stop: f/3.5; 2) Exposure time: 1/2-1/8 s; 3) ISO speed: ISO-500; 4) Exposure bias: -1 step; 5) Focal length: 18 mm; 6) Max aperture: 3.6; 7) Metering mode: Multi-zone; 8) Flash mode: No flash; 9) 35 mm focal length: 27; 10) Color temperature: 4000 K. Subsequently, these images were processed using ImageJ with Java to measure the fluorescence ratios between the control and test lines. To measure magnetic signals, a magnetic assay reader (Magna Bioscience LLC, San Diego, CA, USA) was employed. The magnitude of the magnetic signal captured is in direct correlation with the quantity of magnetic material present in the assay reaction line, and it is presented in terms of relative magnetic units (RMU). More specifically, once the combined scan response curve is calculated and correlated, a scaled value is obtained from the correlated data at each peak position for the control line and all specified test lines. As each analyzer utilizes the same technique for determining these peak values, they are reported in RMU. RMU is an arbitrary scale

relative to each individual analyzer. To ensure consistent magnetic measurement values across analyzers, each analyzer is calibrated against the same magnetic reference standard at the factory. From this calibration standard, a calibration conversion table is computed and stored in the analyzer's permanent memory. Subsequently, after the analyzer computes a control or test line value in RMUs, the value is then converted to a calibrated value and reported as MAR (magnetic measurement value) values.[3] MAR values for any test remain consistent across analyzers. Each dataset was subjected to at least three repeated measurements. Brief operation for the magnetic assay reader is shown below, while the detailed operation can be viewed in the supporting video S1 and S2.

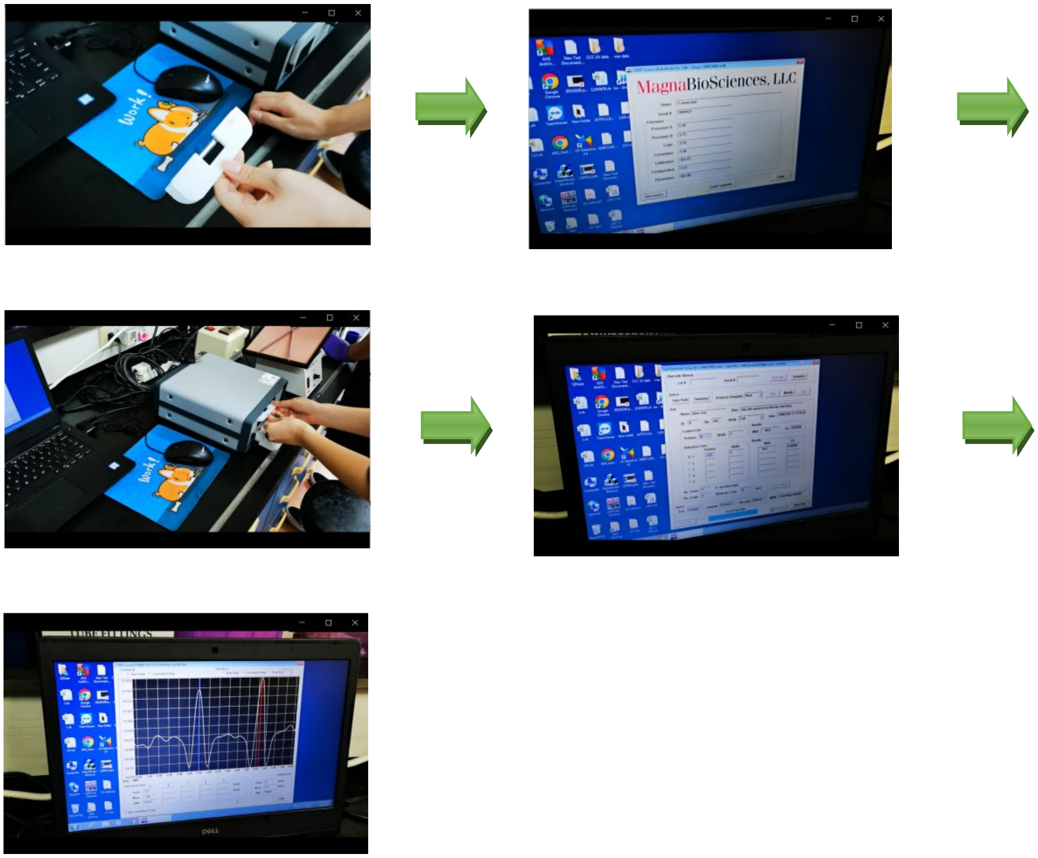

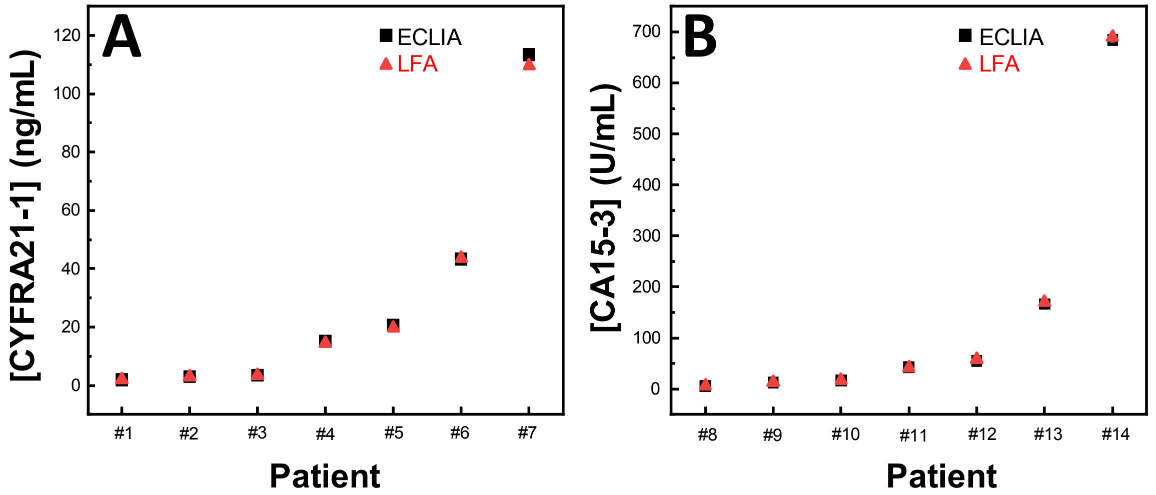

Figure S1. Quantitative determination of CYFRA21-1 and CA15-3 in clinical samples from patients #1-7 with lung cancer and patients #8-14 with breast cancer at different stages. The data obtained from the MNP@AuNP@Pdot-based LFA (red triangles) were compared with that obtained with ECLIA (black squares).

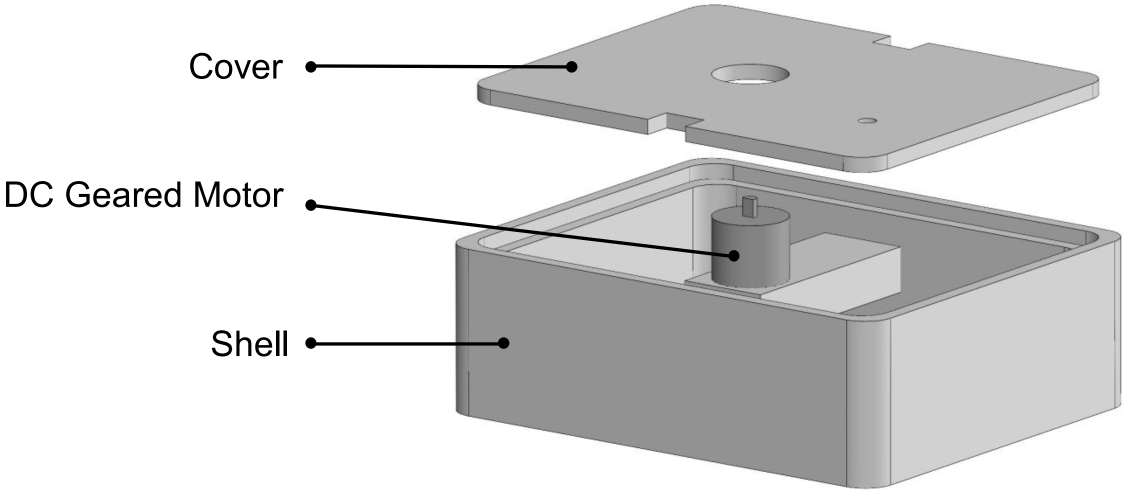

Figure S2. Internal construction of microfluidic centrifugal device.

Supporting Information

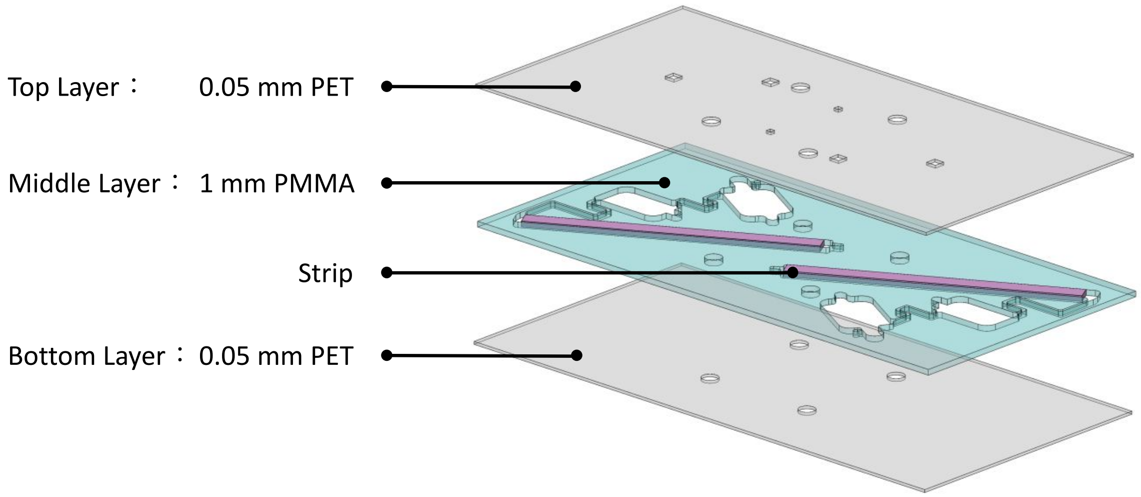

Figure S3. Construction of disposable PMMA microfluidic chip.

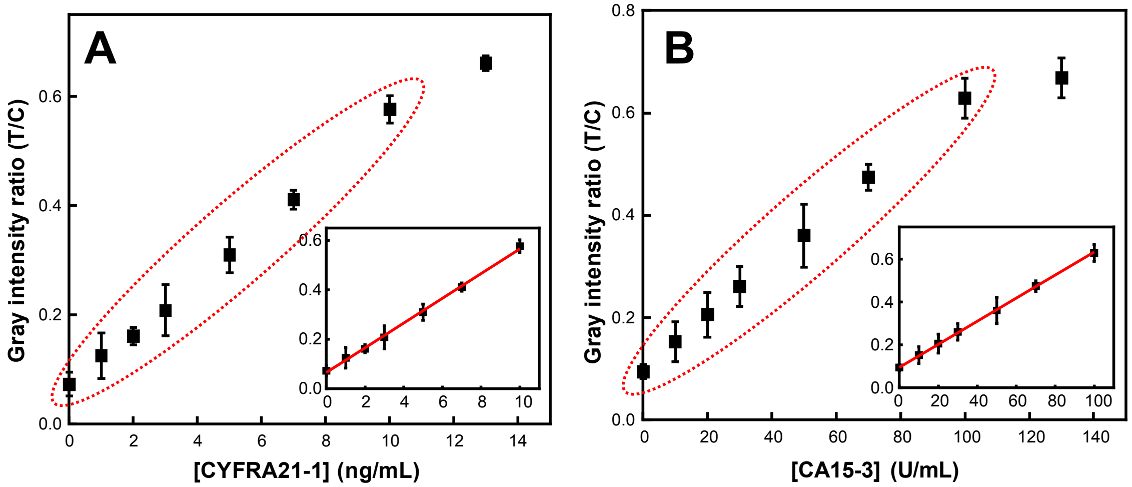

Figure S4. The detection dynamic ranges of (A) CYFRA21-1 and (B) CA15-3 based on colorimetric signals.

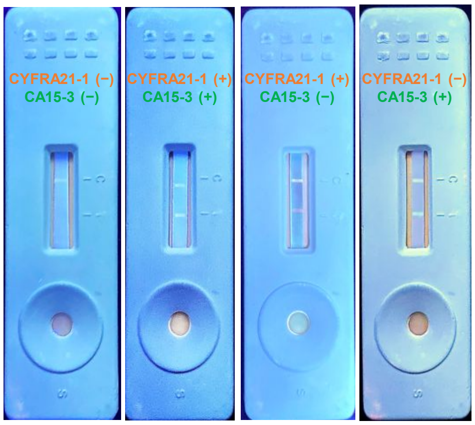

Figure S5. Multiplexed detection of CYFRA21-1 and CA15-3 in serum samples. Photographs of test strips under UV light under ambient conditions.

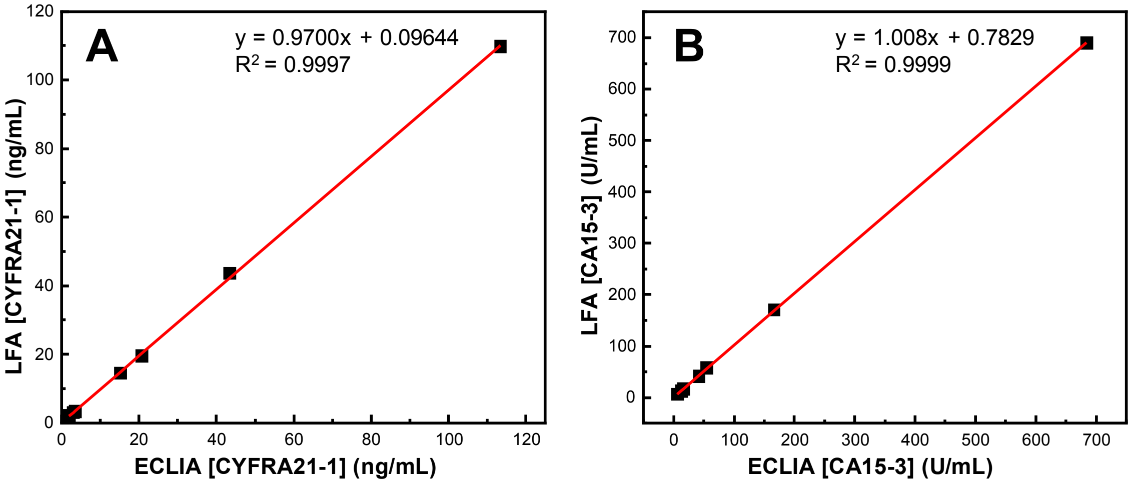

Figure S6. Correlation plots of (A) CYFRA21-1 and (B) CA15-3 values determined using this trimodal LFA and ELISA for clinical samples.

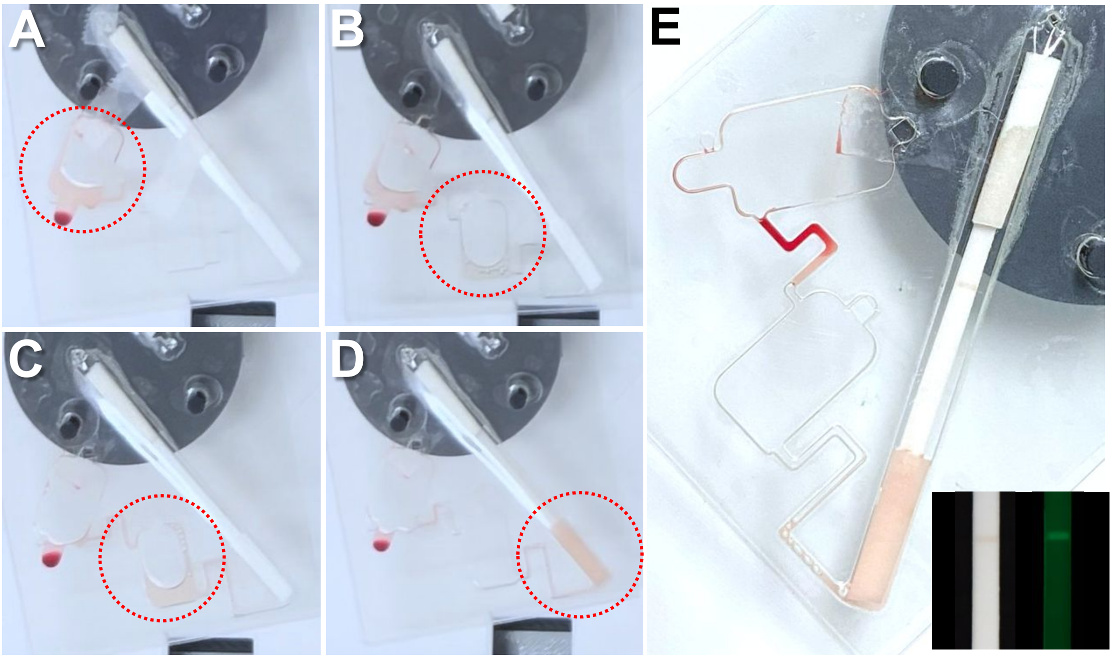

Figure S7. Step-by-step pictures illustrating the operation of the microfluidic device: (A) Separation of red blood cells, (B) addition of probe solution, (C) Mixing of serum and probes, and (D) detection by LFA.

Supporting Information

Table S1. Comparison of CYFRA21-1 detection performance of this trimodal LFA with reported LFAs.

CYFRA21-1	Probe	LOD (ng/mL)	Linear Range (ng/mL)	Multi-modal	Reference
Colorimetry<br>Magnetism<br>Fluorescence	MNP@AuNP@Pdot	1.28<br>0.26<br>0.37	0-10	Triple	This Work
Colorimetry	AuNP	0.55	0.55-500	No	[4]
Magnetism	N/A	N/A	N/A	N/A	N/A
Fluorescence	MNP@Eu^{3+}	0.78	0-1000	No	[5]

Table S2. Comparison of CA15-3 detection performance of this trimodal LFA with reported LFAs.

CA15-3	Probe	LOD (U/mL)	Linear Range (U/mL)	Multi-modal	Reference
Colorimetry<br>Magnetism<br>Fluorescence	MNP@AuNP@Pdot	7.72<br>2.8<br>13	0-100	Triple	This Work
Colorimetry	N/A	N/A	N/A	N/A	N/A
Magnetism	N/A	N/A	N/A	N/A	N/A
Fluorescence	PLGA@MNP	0.09	0-200	Dual	[6]

References:

[1] C.-P. Chen, Y.-C. Huang, S.-Y. Liou, P.-J. Wu, S.-Y. Kuo, Y.-H. Chan, *ACS Appl. Mater. Interfaces* **2014**, 6, 21585.

[2] H.-Y. Liu, P.-J. Wu, S.-Y. Kuo, C.-P. Chen, E.-H. Chang, C.-Y. Wu, Y.-H. Chan, *J. Am. Chem. Soc.* **2015**, 137, 10420.

[3] R. T. LaBorde, B. O'Farrell, *IVD Technol.* **2002**, 8, 36.

[4] L. Xu, S. Wang, Z. Wu, C. Xu, X. Hu, H. Ding, Y. Zhang, B. Shen, Y. Liu, K. Wu, *Front. Bioeng. Biotechnol.* **2022**, 10, 871285.

[5] G. Lin, T. Liu, J. Hou, Z. Ren, J. Zhou, Q. Liang, Z. Chen, W. Dong, Y. Wu, *J. Fluoresc.* **2015**, 25, 361.

[6] B. Zhang, W. Ma, F. Li, W. Gao, Q. Zhao, W. Peng, J. Piao, X. Wu, H. Wang, X. Gong, J. Chang, *Nanoscale* **2017**, 9, 18711.
